# Effects of Methionine Supplementation Levels in Normal or Reduced Protein Diets on the Body Composition and Femur Bone Characteristics of Broilers Challenged with Coccidia

**DOI:** 10.3390/ani14060917

**Published:** 2024-03-16

**Authors:** Guanchen Liu, Venkata Sesha Reddy Choppa, Milan Kumar Sharma, Hanseo Ko, Janghan Choi, Woo Kyun Kim

**Affiliations:** Department of Poultry Science, University of Georgia, Athens, GA 30602, USA; gl16770@uga.edu (G.L.); venkataseshareddy.choppa@uga.edu (V.S.R.C.); milan.sharma@uga.edu (M.K.S.); hsko@uga.edu (H.K.); choij@uga.edu (J.C.)

**Keywords:** methionine, broiler, coccidiosis, bone health, DEXA, micro-CT

## Abstract

**Simple Summary:**

Coccidiosis has been documented to adversely affect the bone quality of broilers. While the influence of minerals and vitamins on bone development has been studied, the impact of methionine supplementation on the bone health of broilers facing coccidia challenge remains inadequately explored. This study aimed to provide insights into the effects of varying dietary methionine levels in normal or reduced protein diets on the bone quality of broilers challenged with coccidia, utilizing X-ray scanning techniques. Interestingly, our results showed that increased methionine levels were associated with decreased whole body bone mineral content and density. In the femur bone, higher methionine levels were associated with decreased cortical bone quality, while they improved trabecular bone quality in birds fed reduced protein diets. Overall, this study sheds light on the complex interplay between dietary methionine levels, protein content, and coccidiosis challenge on broiler bone health, providing information to improve the bone health and welfare of birds under coccidia challenge through nutritional interventions.

**Abstract:**

This study investigated the effects of dietary methionine (Met) levels on the bone quality of broilers challenged with coccidia. A total of 600 fourteen-day-old male Cobb500 broilers were gavaged with mixed *Eimeria* spp. and randomly allocated into 10 treatment groups by a 2 × 5 factorial arrangement. Birds received normal protein diets (NCP) or reduced-protein diets (LCP), containing 2.8, 4.4, 6.0, 7.6, and 9.2 g/kg of Met. Data were analyzed via two-way ANOVA and orthogonal polynomial contrast. At 9 days post-inoculation (DPI), whole body bone mineral density (BMD) and bone mineral content (BMC) linearly decreased as Met levels increased (*p* < 0.05). For the femoral metaphysis bone quality at 9 DPI, BMD linearly decreased, and porosity linearly increased as Met levels increased (*p* < 0.05) in the cortical bone. The increased Met levels linearly improved trabecular bone quality in LCP groups (*p* < 0.05) while not in NCP groups. For the femoral diaphysis cortical bone at 6 DPI, LCP groups had higher BMD and BMC than NCP groups (*p* < 0.05). Bone volume linearly increased as Met levels increased in LCP groups (*p* < 0.05) while not in NCP groups. In summary, the results suggested that increased Met levels decreased the cortical bone quality. However, in the context of reduced-protein diets, the increased Met levels improved trabecular bone quality.

## 1. Introduction

Coccidiosis, caused by the protozoan parasite of the genus *Eimeria*, remains a significant threat to poultry health worldwide, leading to substantial economic losses [1]. The infection of coccidia presents multifaceted challenges to broiler health and productivity. Numerous studies have highlighted its detrimental effects on nutrient utilization, intestinal integrity, and the growth performance of birds [2,3,4,5]. On the other hand, previous research has also documented a range of adverse outcomes on bone health by coccidia infection, including reduced bone mineralization leading to greater porosity in cortical bone, diminished bone mineral density, weakened bone strength, and increased trabecular bone resorption [6,7]. These findings underscore the systemic impact of coccidiosis, extending beyond the gastrointestinal tract to affect skeletal development and integrity.

Due to the concerns about antimicrobial resistance and food safety, as well as evolving regulatory restrictions, the broiler production of “no antibiotics ever” (NAE) or “raised without antibiotics” (RWA) has become more and more prevalent during the past few years [8,9]. This transition underscores the imperative to explore alternative strategies to mitigate the impact of coccidiosis. In addition to investigating the effects of feed additives, such as probiotics, prebiotics, and phytogenic compound supplementations [10], exploring the optimal nutrient requirements for birds under challenged conditions is also vital to improve the health and performance of these animals [11,12,13,14].

Methionine (Met), as the first limiting amino acid in poultry diets, serves not only as the vital building block of proteins but also possesses many functional roles [15,16,17,18]. As previous studies showed, the detrimental effects caused by coccidiosis on bone development were attributed to the elevated oxidative stress and imbalance between osteoclast and osteoblast activities [19,20]. Extra dietary levels of Met could potentially alleviate the impacts of coccidia challenge on bone health due to its potent antioxidant capacity and immune modulatory effects [21,22,23,24]. The reduction in dietary crude protein (CP) content has been advocated for its numerous advantages, including cost reduction, decreased nitrogen excretion, and improved litter quality [2,25,26,27,28,29].

Despite the recognized benefits of adjusting dietary CP levels and increasing Met supplementation, limited research has investigated the specific effects of varying Met levels on bone health in broilers subjected to coccidia challenge. Thus, the present study aimed to address this gap by examining the influence of different Met supplementation levels, within normal or reduced-protein diets, on the bone characteristics of broilers under coccidia challenge.

## 2. Materials and Methods

All animal experiment procedures used in this study were approved by the Institutional Animal Care and Use Committee of the University of Georgia (A2021 12-012).

### 2.1. Experimental Design and Bird Husbandry

The same starter diet that met the breeder’s nutrient recommendations [30] was fed to 600 male Cobb broilers from days 0 to 14. On day 14, all birds were orally gavaged with 25,000 oocysts of *E. maxima*, 25,000 oocysts of *E. tenella*, and 125,000 oocysts of *E. acervulina* to induce coccidiosis infection. On day 14, the birds were randomly allocated into 10 treatment groups with 5 replicates per treatment and 12 birds per replicate. The treatment groups followed a 2 × 5 factorial arrangement. In the normal protein groups (NCP), the diets contained 20% crude protein (CP). As for the low-protein groups (LCP), the diets contained 17% CP, with a 15% reduction from the recommended protein level. The amino acid levels, excluding Met, in the NCP group were formulated to meet the breeder’s recommendation, whereas in the LCP group, they were also reduced by 15%. In both groups, DL-Met was supplemented to achieve Met levels of 2.8, 4.4, 6.0, 7.6, and 9.2 g/kg of the diets, designated as Met 2.8, Met 4.4, Met 6.0, Met 7.6, and Met 9.2, respectively. The feedstuffs and chemical composition of the diets are shown in Table 1. Twelve birds were reared in each battery cage throughout the experimental period from day 0 to d 23. The metallic wire mesh cage was equipped with trough drinker and feeder to provide the birds with ad libitum feed and water. The dimensions of battery cage were 70 cm in length, 60 cm in width, and 30 cm in height. The temperature was set at 33 °C on day 0 and then decreased by 3 °C each week according to the Cobb 500 Broiler Management Guide [31]. The lighting regime was maintained at 22 h of light and 2 h of darkness throughout the experiment period.

### 2.2. Dual-Energy X-ray Absorptiometry for Body Composition Analysis

The body composition analysis was performed by a GE whole body dual-energy X-ray absorptiometry (DEXA) scanner (GE Healthcare, Chicago, IL, USA) according to the previously described method [32]. At 9 days post-inoculation (DPI), before initiating the scanning analysis, a quality assurance program was executed, utilizing a phantom standard to ensure the accurate calibration of the equipment. Subsequently, one bird per cage was euthanized and carefully positioned chest up on the scanner, ensuring no overlap of body parts among the birds. The scanning mode was adjusted for small animals. After the scanning process, the region of interest was determined for each individual bird to measure whole body bone density, mineral content, fat percentage, and lean tissue weight.

### 2.3. Microtomography Scanning for Microstructural Analysis of the Femur Bone

**Figure 1:**
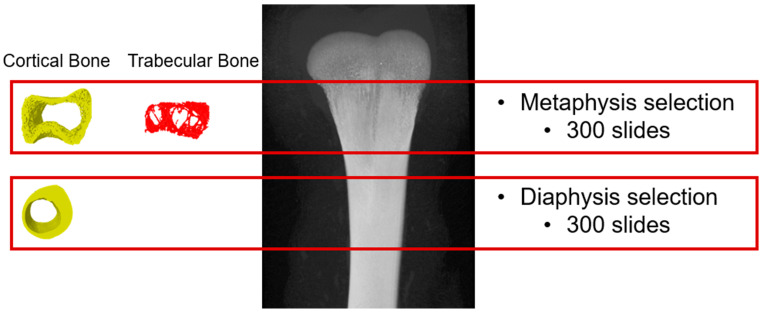
The region of interest selection for the analysis. The same ROI was selected for all the samples. For the metaphysis, 300 slides (7.5 mm) were selected and for the diaphysis, 300 slides (7.5 mm) were selected.

At 6 and 9 days post-inoculation, another bird from each cage was euthanized for femur bone collection. After removal of muscles and soft tissues, the bones were wrapped in wet cheesecloth and held in a low-density 50 mL tube to prevent any random movement during the scanning. Prior to the scanning, the alignment test and flat-field correction were performed for calibration. The samples were scanned by the Skyscan Micro-CT scanner (Skyscan 1275; Bruker MicroCT, Billerica, MA, USA) for 3D image acquisition. The X-ray source was configured to operate at 75 kV and 133 μA, with a 0.5 mm aluminum filter employed to reduce beam-hardening effects. The pixel size remained constant at 25 μm. Scanning was conducted over a 180° rotation with a rotation angle of 0.4°, and 4 images were captured per rotation. The captured 2D images were then reconstructed by the N-Recon program (Bruker MicroCT, Billerica, MA, USA) to generate a 3D model. The resultant model was realigned to a vertical position using the Data Viewer program (Bruker MicroCT, Billerica, MA, USA) to ensure the consistency of the regions of interest (ROIs) selection. The ROI was selected in the CTAn program (Bruker MicroCT, Billerica, MA, USA). The distal metaphysis of 300 slides (7.5 mm in height) and the diaphysis of 300 slides (7.5 mm in height) were selected for analysis (Figure 1). The selected ROI was then processed for cortical and trabecular bone separation according to previously described procedures [33], and the parameters listed and described [34] in Table 2 were measured. To measure bone mineral density (BMD), two phantoms composed of calcium hydroxyapatite with known densities (0.25 and 0.75 g/cm^3^) were scanned using identical settings as those used for bone scanning to generate the standards [35].

### 2.4. Statistical Analysis

The PROC GLM program in SAS software (version 9.4; SAS Institute Inc., Cary, NC, USA) was used for the statistical analysis of the obtained data. The data were analyzed by two-way ANOVA. Post hoc analysis was conducted using the Tukey’s honestly significant difference test when significant differences were detected. Orthogonal polynomial contrasts were utilized to evaluate linear and quadratic trends between the various Met levels and measured parameters. Statistical significance was set at *p* ≤ 0.05.

## 3. Results

### 3.1. Body Composition Analyzed by Dual-Energy X-ray Absorptiometry

**Figure 2:**
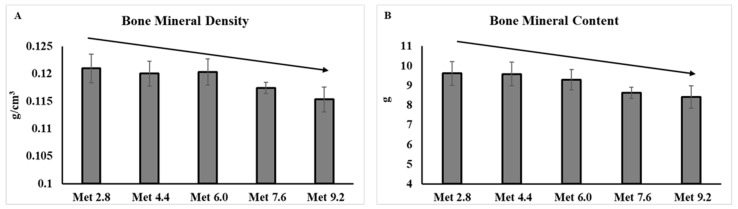
Body composition analyzed by dual-energy X-ray absorptiometry. The error bars represent the SEM values. The black lines with arrowhead represented significant linear or quadratic relationship between parameters and dietary methionine levels. Statistical significance was set at *p* ≤ 0.05. Met, methionine; Met 2.8, dietary Met level = 2.8 g/kg; Met 4.4, dietary Met level = 4.4 g/kg; Met 6.0, dietary Met level = 6.0 g/kg; Met 7.6, dietary Met level = 7.6 g/kg; Met 9.0, dietary Met level = 9.0 g/kg. (**A**), *p*-value: *p*-linear = 0.049. (**B**), *p*-value: *p*-linear = 0.045.

No significant treatment effects were found for the body composition results. However, as the dietary Met levels increased, the whole body BMD, *F*(1,40) = 4.12, *p =* 0.049, and bone mineral content (BMC), *F*(1,40) = 4.27, *p =* 0.045, linearly decreased (Figure 2).

### 3.2. Femur Bone Microstructure Analyzed by Microtomography

#### 3.2.1. Metaphysis Cortical Bone

**Figure 3:**
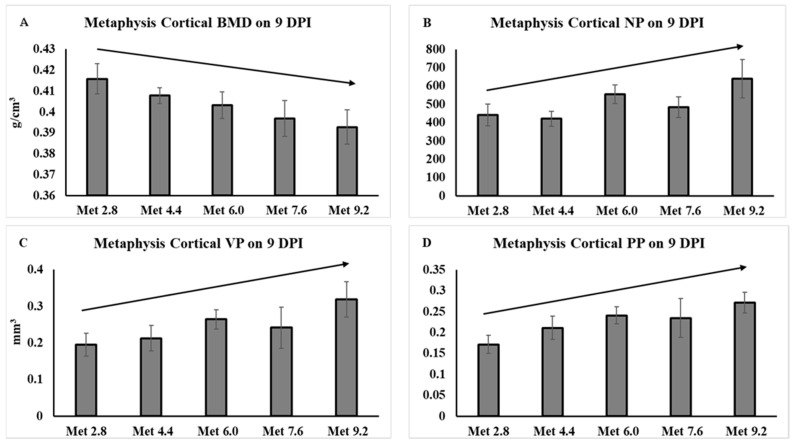
Femoral metaphysis cortical bone characteristics analyzed by microtomography. The error bars represent the SEM values. The black lines with arrowhead represented significant linear or quadratic relationship between parameters and dietary methionine levels. Statistical significance was set at *p* ≤ 0.05. DPI, day post inoculation; BMD, bone mineral density; NP, number of closed pores; VP, volume of closed pores; PP, closed pore percentage; Met, methionine; Met 2.8, dietary Met level = 2.8 g/kg; Met 4.4, dietary Met level = 4.4 g/kg; Met 6.0, dietary Met level = 6.0 g/kg; Met 7.6, dietary Met level = 7.6 g/kg; Met 9.0, dietary Met level = 9.0 g/kg. (**A**), *p*-value: *p*-linear = 0.020. (**B**), *p*-value: *p*-linear = 0.029. (**C**), *p*-value: *p*-linear = 0.037. (**D**), *p*-value: *p*-linear = 0.023.

No significant treatment effects were found for the parameters measured for the femoral metaphysis cortical bone on 6 DPI. At 9 DPI, the BMD linearly decreased as Met levels increased, *F*(1,40) = 5.95, *p =* 0.020, and the number of pores (NP), *F*(1,40) = 5.14, *p =* 0.029, volume of pores (VP), *F*(1,40) = 4.70, *p =* 0.037, and pore percentage (PP), *F*(1,40) = 5.66, *p =* 0.023, linearly increased as Met levels increased (Figure 3).

#### 3.2.2. Metaphysis Trabecular Bone

**Figure 4:**
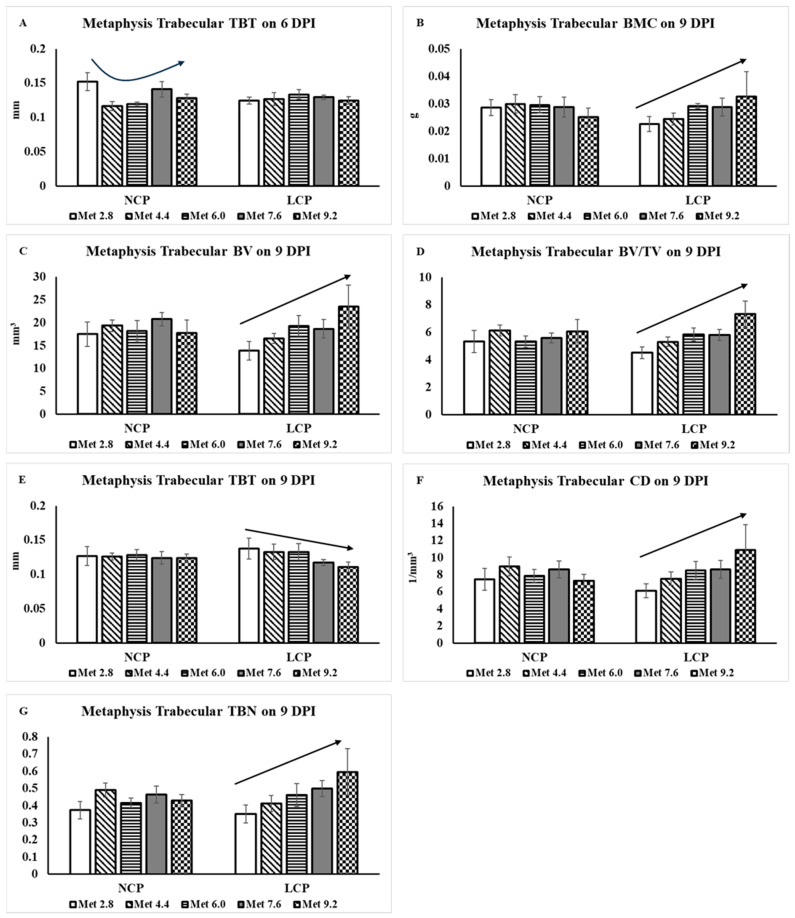
Femoral metaphysis trabecular bone characteristics analyzed by microtomography. The error bars represent the SEM values. Bars without a common letter differ significantly. The black lines with arrowhead represented significant linear or quadratic relationship between parameters and dietary methionine levels. Statistical significance was set at *p* ≤ 0.05. DPI, days post-inoculation; TBT, trabecular thickness; BMC, bone mineral content; BV, bone volume; TV, tissue volume; CD, connectivity density; TBN, trabecular number. Met, methionine; NCP, normal protein diet; LCP, reduced-protein diet; Met 2.8, dietary Met level = 2.8 g/kg; Met 4.4, dietary Met level = 4.4 g/kg; Met 6.0, dietary Met level = 6.0 g/kg; Met 7.6, dietary Met level = 7.6 g/kg; Met 9.0, dietary Met level = 9.0 g/kg. (**A**) *p*-value: *p*-quadratic at NCP = 0.030, *p*-quadratic at LCP = 0.399. (**B**) *p*-value: *p*-linear at NCP = 0.493, *p*-linear at LCP = 0.050. (**C**) *p*-value: *p*-linear at NCP = 0.800, *p*-linear at LCP = 0.009. (**D**) *p*-value: *p*-linear at NCP = 0.670, *p*-linear at LCP = 0.003. (**E**) *p*-value: *p*-linear at NCP = 0.805, *p*-linear at LCP = 0.041. (**F**) *p*-value: *p*-linear at NCP = 0.651, *p*-linear at LCP = 0.004. (**G**) *p*-value: *p*-linear at NCP = 0.852, *p*-linear at LCP = 0.011.

At 6 DPI, the trabecular thickness (TBT) decreased initially and then increased following a quadratic trend as Met levels increased in the NCP groups, *F*(1,40) = 5.03, *p* = 0.030, while not in the LCP groups (Figure 4). However, the treatment effects were not significant for other measured parameters. At 9 DPI, no treatment effects were observed for the tissue volume (TV) and trabecular separation (TBS). The BMC, *F*(1,40) =4.10, *p =* 0.050, bone volume (BV), *F*(1,40) = 7.51, *p =* 0.009, BV/TV ratio, *F*(1,40) = 10.3, *p =* 0.028, trabecular number (TBN), *F*(1,40) = 9.28, *p* = 0.004, and connectivity density (CD), *F*(1,40) = 7.19, *p =* 0.011, linearly increased as Met levels increased in the LCP groups while not in the NCP groups. The TBT linearly decreased as Met levels increased in the LCP groups, *F*(1,40) = 4.48, *p =* 0.041, while not in the NCP groups.

#### 3.2.3. Diaphysis Cortical Bone

**Figure 5:**
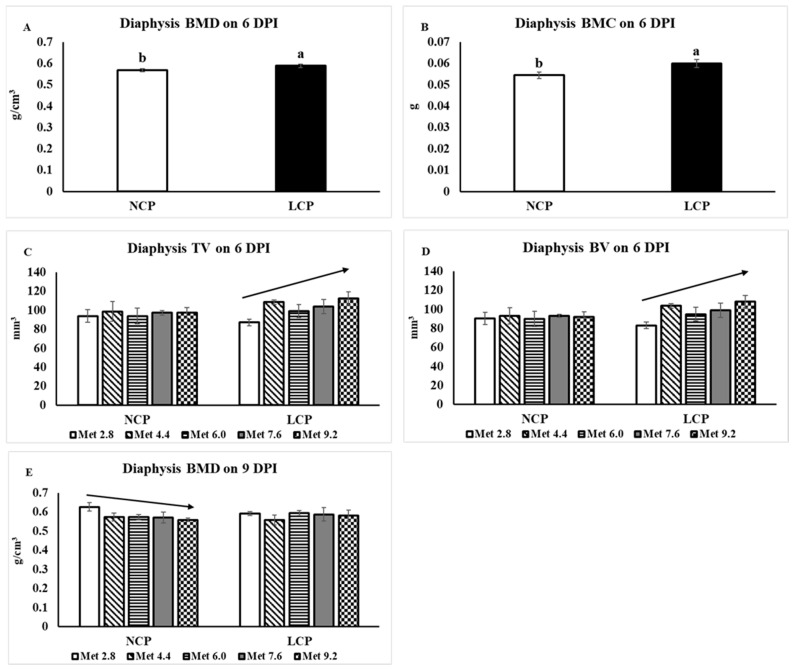
Femoral diaphysis cortical bone characteristics analyzed by microtomography. The error bars represent the SEM values. The black lines with arrowhead represented significant linear or quadratic relationship between parameters and dietary methionine levels. Statistical significance was set at *p* ≤ 0.05. DPI, day post inoculation; BMD, bone mineral density; BMC, bone mineral content; TV, tissue volume; BV, bone volume; Met, methionine; NCP, normal protein diet; LCP, reduced protein diet; Met 2.8, dietary Met level = 2.8 g/kg; Met 4.4, dietary Met level = 4.4 g/kg; Met 6.0, dietary Met level = 6.0 g/kg; Met 7.6, dietary Met level = 7.6 g/kg; Met 9.0, dietary Met level = 9.0 g/kg. (**A**) *p*-value: *p*-protein = 0.048. (**B**) *p*-value: *p*-protein = 0.026. (**C**) *p*-value: *p*-linear at NCP = 0.801, *p*-linear at LCP = 0.033. (**D**) *p*-value: *p*-linear at NCP = 0.856, *p*-linear at LCP = 0.023. (**E**) *p*-value: *p*-linear at NCP = 0.039, *p*-linear at LCP = 0.906.

At 6 DPI, the BMD, *F*(1,40) = 4.18, *p =* 0.048, and BMC, *F*(1,40) = 5.32, *p =* 0.026, were both significantly higher in the LCP groups compared to the NCP groups (Figure 5). The TV, *F*(1,40) = 4.86, *p* = 0.033, and BV, *F*(1,40) = 5.55, *p* = 0.023, linearly increased as Met levels increased in the LCP groups while not in the NCP groups. No significant treatment effects were observed for other parameters. At 9 DPI, the BMD linearly decreased as Met levels increased in the NCP groups, *F*(1,40) = 4.57, *p* = 0.039, while not in the LCP groups.

## 4. Discussion

This study employed DEXA and Micro-CT scanning techniques to evaluate various parameters related to broiler bone health, shedding light on this critical aspect regarding both the nutrient requirements of broilers under coccidia challenge and their welfare. Overall, while no significant treatment effects were observed for body composition, notable alterations were detected in the whole body BMD, BMC, and bone microstructure parameters in the femur bone.

It is interesting to find that increasing Met levels in the diet decreased the whole body BMD and BMC, regardless of the type of diet. This implies that a high Met intake may have a negative impact on the overall bone quality and mass. This was further evidenced as we analyzed the femur bone microstructure and found that increasing Met levels in the diet decreased the BMD of the femoral metaphysis cortical bone and increased the porosity of the cortical bone, especially at 9 DPI. Elevated porosity is commonly associated with diminished bone strength and stiffness, increasing susceptibility to fractures under stress or impact [35,36,37]. Moreover, increased porosity can compromise the bone’s role as a reservoir of essential minerals, such as calcium and phosphorus, which are vital for numerous metabolic processes in the body [38], as evidenced by the observed decrease in BMD. This reduction in bone quality in the metaphysis cortical bone could be due to the increased accumulation of homocysteine (Hcy) caused by increased dietary Met levels, as multiple studies have demonstrated [39,40,41]. Hcy has been recognized as a risk factor for bone disease development [42,43,44]. As previous studies have reported, increased Hcy levels were associated with elevated levels of matrix metalloproteinases that contribute to bone matrix degradation [45] and increased bone-resorbing osteoclast activity [44,46]. This hypothesis needs to be investigated in future studies to fully understand the mechanisms before this decreased cortical bone quality.

More interestingly, the effects of increased Met levels exerted different effects on the metaphysis trabecular microstructures. While the trabecular bone quality was not affected by the treatments in the NCP groups, it was improved in the LCP groups as Met levels increased. As previously mentioned, trabecular bone is more responsive to immune responses [47,48,49], and one previous study showed that the bone mineral loss during coccidiosis occurred more in the trabecular bone [7], so it is plausible to hypnotize that instead of being accumulated as Hcy, the extra Met supplementation exerted its functional roles, such as antioxidant capacity, immunomodulatory effects, and collagen synthesis [15,50], especially in the LCP groups where the supply of other essential and non-essential amino acids was also reduced. Consequently, trabecular bone quality may have improved, as evidenced by the increased trabecular number and connectivity density.

It is also intriguing to observe that the reduced-protein diet improved BMD and BMD in the femoral diaphysis. Some prior studies have suggested that high-protein diets could be detrimental to bone health due to increased urinary calcium excretion resulting from the metabolic acidity of protein catabolism [51,52,53]. In acidic conditions, the activities of osteoclasts are increased while the activities of bone-forming osteoblasts are suppressed [54,55]. However, this point of view remains debatable, with evidence also suggesting that increased protein intake can be beneficial for bone health [56,57]. It is essential to recognize that these studies have predominantly focused on human subjects, with limited evidence available in poultry, especially for broilers under conditions of coccidiosis challenge. Further studies are needed to further investigate this question in poultry.

## 5. Conclusions

In conclusion, the findings of the current study suggest that in broilers under coccidia challenge, increased dietary Met levels were associated with a decrease in cortical bone quality parameters. However, in the context of reduced-protein diets, such an increase in Met levels conversely improved trabecular bone quality. The findings of this study underscored the complex interplay between dietary components, particularly Met, and bone health in broilers under coccidiosis challenge, highlighting the importance of considering dietary interventions for optimizing skeletal integrity and overall welfare in poultry production.

## Figures and Tables

**Table 1:** Ingredient formulation and nutrient and energy composition (g/kg) of experimental diets.

	NCP ^1^					LCP				
Ingredient	Met 2.8	Met 4.4	Met 6.0	Met 7.6	Met 9.2	Met 2.8	Met 4.4	Met 6.0	Met 7.6	Met 9.2
Corn	692	692	692	692	692	730	730	730	730	730
Soybean Meal	248	248	248	248	248	221	221	221	221	221
Soybean Oil	3.00	3.00	3.00	3.00	3.00	1.10	1.10	1.10	1.10	1.10
Common Salt	3.50	3.50	3.50	3.50	3.50	3.50	3.50	3.50	3.50	3.50
Limestone	11.8	11.8	11.8	11.8	11.8	11.9	11.9	11.9	11.9	11.9
Dicalcium Phosphate	7.90	7.90	7.90	7.90	7.90	8.00	8.00	8.00	8.00	8.00
Vitamin Premix ^2^	1.00	1.00	1.00	1.00	1.00	1.00	1.00	1.00	1.00	1.00
Mineral Premix ^3^	0.80	0.80	0.80	0.80	0.80	0.80	0.80	0.80	0.80	0.80
DL-Methionine	0.00	1.60	3.20	4.80	6.40	0.10	1.70	3.30	4.90	6.50
L-Lysine HCl	4.10	4.10	4.10	4.10	4.10	2.70	2.70	2.70	2.70	2.70
L-Glutamate	8.00	8.00	8.00	8.00	8.00					
Threonine	1.30	1.30	1.30	1.30	1.30	0.60	0.60	0.60	0.60	0.60
Arginine	1.10	1.10	1.10	1.10	1.10	0.10	0.10	0.10	0.10	0.10
L-Cystine						0.10	0.10	0.10	0.10	0.10
Isoleucine	0.20	0.20	0.20	0.20	0.20					
Glycine	10.0	9.20	8.40	7.60	6.80	5.00	4.30	3.50	2.60	1.80
Sand	7.60	6.80	6.00	5.20	4.60	14.5	13.6	12.80	12.10	11.30
Total	1000	1000	1000	1000	1000	1000	1000	1000	1000	1000
Calculated Nutrients (g/kg) and Energy										
Protein	200	200	200	200	200	170	170	170	170	170
ME, kcal/kg	3030	3030	3030	3030	3030	3030	3030	3030	3030	3030
Ca	7.00	7.00	7.00	7.00	7.00	7.00	7.00	7.00	7.00	7.00
Available P	2.90	2.90	2.90	2.90	2.90	2.90	2.90	2.90	2.90	2.90
Lysine	11.2	11.2	11.2	11.2	11.2	9.50	9.50	9.50	9.50	9.50
Methionine	2.80	4.40	6.00	7.60	9.20	2.80	4.40	6.00	7.60	9.20
TSAA	5.30	6.90	8.50	10.1	11.7	5.30	6.90	8.50	10.1	11.7
Threonine	7.30	7.30	7.30	7.30	7.30	6.20	6.20	6.20	6.20	6.20
Arginine	11.8	11.8	11.8	11.8	11.8	10.0	10.0	10.0	10.0	10.0
Analyzed Amino Acids, g/kg										
Protein	195	199	211	211	204	177	182	176	181	176
Lysine	13.1	12.5	12.7	13.0	13.2	10.5	11.4	11.4	11.6	11.3
Methionine	2.90	4.50	5.90	7.80	8.90	2.90	4.50	6.30	7.20	8.80
TSAA	5.90	7.40	9.10	10.9	11.5	5.80	7.60	9.20	10.3	11.8
Threonine	7.60	7.60	7.70	7.60	7.70	6.50	7.10	7.00	7.10	7.00
Arginine	12.1	11.7	11.6	12.4	12.5	10.1	11.3	11.0	11.5	10.6
Glutamate	39.2	37.3	37.3	39.3	39.2	30.2	32.7	32.2	33.3	31.5
Amino Acids to Lysine Ratios										
Lysine	100	100	100	100	100	100	100	100	100	100
Methionine	22.2	34.1	46.5	59.8	67.4	27.1	39.5	54.8	62.1	77.9
TSAA	45.2	56.3	71.7	83.8	90.5	54.8	66.7	80.7	88.8	104
Threonine	57.9	60.4	60.6	58.3	58.0	61.4	62.3	60.9	61.2	61.5
Arginine	92.7	89.3	91.3	95.7	94.7	96.2	99.1	96.5	98.7	93.8
Glutamate	300	286	294	303	297	288	287	282	287	278

^1^ NCP, normal protein diet with 20% crude protein content; LCP, reduced-protein diet with 17% crude protein content; Met 2.80, diet containing 2.80 g/kg of methionine; Met 4.40, diet containing 4.40 g/kg of methionine; Met 6.00, diet containing 6.00 g/kg of methionine; Met 7.60, diet containing 7.60 g/kg of methionine; Met 9.20, diet containing 9.20 g/kg of methionine. ^2^ Vitamin premix: Supplemented per kg of diet: thiamin mononitrate, 2.4 mg; nicotinic acid, 44 mg; riboflavin, 4.4 mg; D-Ca pantothenate, 12 mg; vitamin B12 (cobalamin), 12.0 g; pyridoxine HCl, 4.7 mg; D-biotin, 0.11 mg; folic acid, 5.5 mg; menadione sodium bisulfite complex, 3.34 mg; choline chloride, 220 mg; cholecalciferol, 27.5 g; transretinyl acetate, 1892 g; *α* tocopheryl acetate, 11 mg; ethoxyquin, 125 mg. ^3^ Mineral premix: Supplemented as per kg of diet: manganese (MnSO_4_·H_2_O), 60 mg; iron (FeSO_4_·7H_2_O), 30 mg; zinc (ZnO), 50 mg; copper (CuSO_4_·5H_2_O), 5 mg; iodine (ethylene diaminedihydroiodide), 0.15 mg; selenium (NaSeO_3_), 0.3 mg.

**Table 2:** Definition and description of microtomography measured parameters.

Parameters (Abbreviation)	Description	Standard Unit
Bone mineral density (BMD)	Measure the bone mineral content per unit of volume	g/cm^3^
Bone mineral content (BMC)	Measure the bone mineral content of the tissue. The value is calculated by BMD × TV	g
Tissue volume (TV)	The volume of the entire region of interest, including pores and cavity inside the bone	mm^3^
Bone volume (BV)	Volume of the bone segments	mm^3^
Bone volume fraction (BV/TV)	Ratio of the bone volume to the tissue volume	%
Number of closed pores (NP)	Number of closed pores within the bone segments	
Volume of closed pores (VP)	Total volume of the closed pores	mm^3^
Closed pore percentage (PP)	The volume of closed pores as a percentage of the bone volume	%
Trabecular number (TBN)	The average number of trabeculae per unit length	1/mm
Trabecular thickness (TBT)	Mean thickness of trabeculae, measured using 3D methods	mm
Trabecular separation (TBS)	Mean distance between trabeculae, measured using 3D methods	mm
Connectivity density (CD)	A measure of the degree of connectivity of trabeculae normalized by tissue volume	1/mm^3^

## Data Availability

Data generated or analyzed during this study are included in the published article and available from the corresponding author upon reasonable request.
